# Lactate and CO_2_-derived parameters are not predictive factors of major postoperative complications after cardiac surgery with cardiopulmonary bypass: a diagnostic accuracy study

**DOI:** 10.3389/fcvm.2025.1504431

**Published:** 2025-04-11

**Authors:** Xiao-Fen Zhou, Han Chen, Jun Ke, Shi-Rong Lin, Ting-Feng Huang, Bing-Ying Chen, Xin-Da Jiang, Feng Chen

**Affiliations:** ^1^ Shengli Clinical Medical College of Fujian Medical University, Fuzhou, Fujian, China; ^2^ The Fourth Department of Critical Care Medicine, Fuzhou University Affiliated Provincial Hospital, Fuzhou, Fujian, China; ^3^ Fujian Provincial Key Laboratory of Emergency Medicine, Fuzhou, Fujian, China; ^4^ Fujian Emergency Medical Center, Fuzhou, Fujian, China; ^5^ Department of Emergency, Fuzhou University Affiliated Provincial Hospital, Fuzhou, Fujian, China

**Keywords:** lactate, pv-aCO_2_/Ca-vO_2_, cv-aCO_2_/Ca-vO_2_, postoperative complications, diagnostic accuracy study

## Abstract

**Purpose:**

This study aimed to compare the performance of lactate and CO_2_-derived parameters in predicting major postoperative complications (MPC) after cardiac surgery with cardiopulmonary bypass.

**Methods:**

Lactate and CO_2_-derived parameters, including the venous-arterial difference in CO_2_ partial pressure (Pv-aCO_2_), the venous-arterial difference in CO_2_ partial pressure to arterial-venous O_2_ content ratio (Pv-aCO_2_/Ca-vO_2_), and the venous-arterial difference in CO_2_ content to arterial-venous O_2_ content ratio (Cv-aCO_2_/Ca-vO_2_) at ICU admission, 3 h, 6 h, and 12 h later were collected. Receiver-operating characteristics (ROC) curve analysis was carried out to assess the predictive performance. Univariate and multivariate logistic regression analyses were performed to identify independent predictors of MPC.

**Results:**

MPC occurred in 77 (54.2%) of 142 patients. No significant difference was observed between the MPC and no-MPC groups regarding lactate and CO_2_-derived parameters. The area under the curves (AUCs) were 0.532 (0.446–0.616) for lactate, 0.559 (0.473–0.642) for Pv-aCO_2_, 0.617 (0.532–0.697) for Pv-aCO_2_/Ca-vO_2_, and 0.625 (0.540–0.705) for Cv-aCO_2_/Ca-vO_2_, respectively, and there was no significant difference between the parameters. In the *post-hoc* analysis, all parameters' AUCs were lower than 0.75 in predicting acute renal failure, and there was no significant difference between these parameters. Cv-aCO_2_/Ca-vO_2_ at 12 h yielded the highest AUC of 0.853 (0.784–0.907) in predicting mortality and the highest AUC of 0.808 (0.733–0.869) in predicting delirium. In multivariate analysis, hypertension, surgery duration, and PaO_2_/FiO_2_ were identified as independent predictors of MPC, while lactate and CO_2_-derived parameters lost statistical significance after adjustment for covariates.

**Conclusions:**

Lactate and CO_2_-derived parameters cannot be used as reliable indicators to predict the occurrence of MPC after cardiopulmonary bypass. Instead, traditional clinical factors such as hypertension, extended surgical duration, and impaired oxygenation emerged as the most reliable risk indicators.

## Study registration

The study was registered on January 26th, 2020, at the Chinese Clinical Trial Registry (ChiCTR2000029365). URL of trial registry record: https://www.chictr.org.cn/bin/project/edit?pid=48744.

## Introduction

The prompt response to tissue hypoxia following surgery is essential in preventing organ dysfunction and related complications. Biomarkers such as lactate have been proposed for the detection of tissue hypoxia (1–3). However, the presence of cardiopulmonary bypass (CPB) during cardiac surgery can complicate the interpretation of tissue hypoxia. Postoperative hyperlactatemia occurs in approximately 10%–20% of cardiac surgery patients, which may reflect either transient tissue hypoxia due to CPB (which is usually a benign phenomenon) or sustained tissue hypoxia resulting from postoperative circulatory failure (4). While numerous studies have established a link between elevated lactate levels and adverse outcomes post-cardiac surgery, the association is not definitive as elevated lactate levels are not always indicative of sustained tissue hypoxia and anaerobic metabolism (4, 5).

A series of parameters derived from CO₂ have emerged as alternative biomarkers for detecting tissue hypoxia and anaerobic metabolism. These include the venous-arterial difference in CO₂ partial pressure (Pv-aCO₂ or PCO₂ gap), the venous-arterial difference in CO₂ partial pressure to arterial-venous O₂ content ratio (Pv-aCO₂/Ca-vO₂), and the venous-arterial difference in CO₂ content to arterial-venous O₂ content ratio (Cv-aCO₂/Ca-vO₂) (6–9). Pv-aCO₂ primarily reflects the adequacy of cardiac output, as CO₂ accumulates in tissues when perfusion is inadequate, resulting in elevated venous CO₂. However, Pv-aCO₂ alone lacks specificity as a marker of tissue hypoxia and anaerobic metabolism (10). In contrast, both Pv-aCO₂/Ca-vO₂ and Cv-aCO₂/Ca-vO₂ ratios serve as surrogates for the respiratory quotient (RQ)—the ratio of CO₂ production to O₂ consumption—which increases above 1.0 during anaerobic metabolism (11). While the relationship between CO₂ content and CO₂ partial pressure follows a nearly linear relationship under physiological conditions (Pv-aCO₂ = *κ* × Cv-aCO₂, where *κ* represents the relationship factor), this linearity can be disrupted during metabolic acidosis, which is common after cardiac surgery (12–15). This potential Pv-aCO₂-Cv-aCO₂ decoupling has led to questions about whether Pv-aCO₂/Ca-vO₂ and Cv-aCO₂/Ca-vO₂ are truly interchangeable in clinical settings, particularly in cardiac surgery patients where acid-base disturbances are frequent. While these CO₂-derived parameters have been increasingly employed to identify low-flow states and guide resuscitation in septic shock, their clinical utility in cardiac surgery patients remains inadequately explored.

The CO_2_-derived parameters mentioned above, such as Pv-aCO_2_, Cv-aCO_2_/Ca-vO_2_, and Pv-aCO_2_/Ca-vO_2_, are increasingly employed to identify the low-flow status or anaerobic metabolism in septic shock for guiding fluid resuscitation (16, 17). However, limited investigation has been conducted on their application in patients undergoing cardiac surgery. This study aims to assess the efficacy of lactate and CO_2_-derived parameters in predicting major postoperative complications (MPC) following cardiac surgery with CPB.

## Methods

### Study design and population

The present study is a prospective, single-center diagnostic accuracy study involving patients undergoing elective cardiac surgery. The study protocol and consent forms were approved by the Ethics Committee of Fujian Provincial Hospital (Approval # K2020-01-022). The study was registered on January 26th, 2020, at the Chinese Clinical Trial Registry (ChiCTR2000029365). Informed consent was obtained from all subjects and/or their legal guardians. The study protocol was published on October 1st, 2021 (18). The major change in the protocol was that we also compared the performance of lactate and CO_2_-derived parameters in predicting mortality, acute kidney injury (AKI), and delirium, respectively, which are the potential consequences of tissue hypoxia.

Patients who received cardiac surgery with CPB were screened. The inclusion criteria were (1) Age ≥ 18; (2) Elective cardiac surgery with CPB; (3) Admitted into the cardiosurgical intensive care unit (ICU) after the operation; (4) With an arterial and a central venous catheter in place. Exclusion criteria were: (1) Known acute or chronic kidney disease according to international diagnostic standards (19–21); (2) Known hepatic insufficiency prior to surgery; (3) Malpositioning of the central venous catheter confirmed by chest x-ray; (4) History of alcohol abuse; (5) Pregnant; (6) Unwilling to provide consent.

### Data collection and follow-up

Baseline characteristics were collected, including demographic data, medical history, preoperative ejection fraction, and the New York Heart Association (NYHA) functional classification. Intraoperative data were also recorded, including the type of surgery, duration of operation, CPB and aortic clamping, transfusion, fluid balance, and the doses of vasopressors and inotropic agents. The sequential organ failure assessment score (SOFA) and Acute Physiology and Chronic Health Evaluation (APACHE) II score at enrollment and the daily highest score during the follow-up period were recorded. Arterial and venous blood samples were collected at ICU admission (T_0_), and 3 h (T_3_), 6 h (T_6_), and 12 h (T_12_) after admission for the measurement of lactate and the calculation of CO_2_-derived parameters, by using the bedside blood gas analyzer (GEM 3500, Instrumentation Laboratory Co., MA, USA). CO_2_ content was calculated according to the Douglas formula (22), formulas were also provided in our published protocol (18) and the Supplementary File.

Patients were followed-up for seven days starting from the time of ICU admission. MPC was recorded, as previously defined in the protocol (18). Briefly, we defined the MPC as any of the following conditions: (1) AKI; (2) acute respiratory distress syndrome (ARDS) or respiratory failure; (3) cardiac arrest; (4) neurological dysfunction (including delirium); (5) myocardial infarction; (6) circulatory failure; (7) abdominal compartment syndrome; (8) reoperation; (9) death from any cause. Delirium was assessed daily during the 7-day follow-up period using the Confusion Assessment Method for the Intensive Care Unit (CAM-ICU), a validated tool for detecting delirium in critically ill patients. Assessments were performed by trained ICU nurses or physicians (23). The following postoperative data were also collected: the duration of mechanical ventilation and length of ICU stay; results of C-reactive protein, N-terminal pro-B type natriuretic peptide and procalcitonin; and the daily highest vasoactive-inotropic score (VIS) (24).

### Statistical analysis

Perioperative characteristics were compared according to the occurrence or not of MPC. Continuous variables were presented as means and standard deviations (for normal distribution) or medians and interquartile ranges (for non-normal distribution). Student's *t*-test was used for normally distributed variables, and the Mann–Whitney *U*-test was used for non-normally distributed variables. Categorical variables were presented as numbers and percentages and analyzed using the *χ*^2^-test or Fisher's exact tests as appropriate. Changes over time were examined using Friedman RM-ANOVA on ranks where group distribution was not normal or the two-way ANOVA where distribution was normal and variance equal.

Receiver-operating characteristics (ROC) curve analyses were used to assess the predictive performance of lactate and CO_2_-derived parameters. The optimal thresholds for predicting the outcomes were determined by maximizing the Youden index. Sensitivity, specificity, predictive values, and likelihood ratios were calculated. The performance of the ROC curves was compared using DeLong's method (25).

Model 1 employed univariate Cox proportional hazards regression to assess the independent association of each variable with the outcome without covariate adjustment. Model 2 used a forced entry Cox proportional hazards model, retaining key variables and adjusting for additional covariates via forward selection. Model 3 applied forward stepwise multivariate logistic regression, incrementally including variables meeting significance thresholds (*p* < 0.05 for entry, *p* > 0.10 for removal), adjusted for the same covariates as Model 2. A *p*-value < 0.05 (two-tailed) was considered significant. Analyses were performed using SPSS Statistical Software (Ver. 23.0, IBM Co., NY, USA) and MedCalc Statistical Software (to compare ROC curves).

## Results

**Figure 1:**
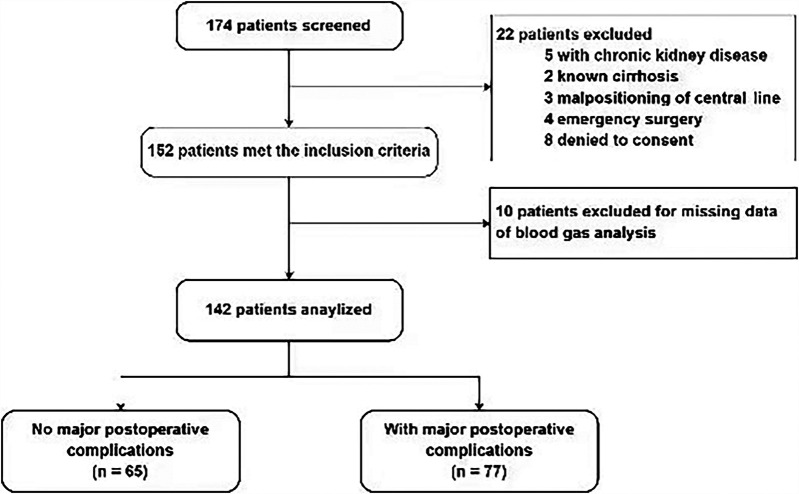
Flowchart showing a step-by-step selection of patients included in the study.

**Figure 2:**
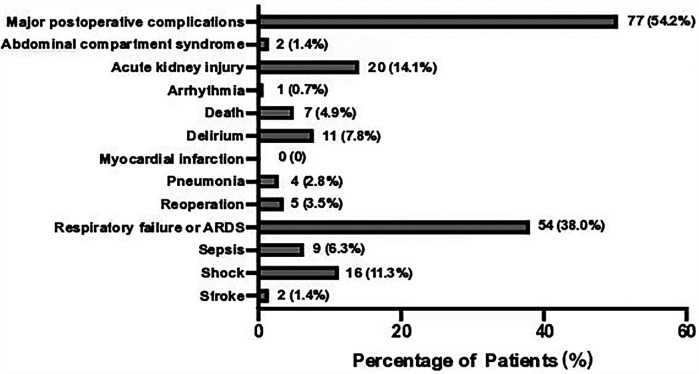
Incidence of major postoperative complications components. Showing the number and percentage of patients. ARDS, acute respiratory distress syndrome.

A total of 142 patients were included between October 1st, 2021 and January 1st, 2024 (Figure 1). MPC occurred in 77 (54.2%) patients. The three leading MPC components were respiratory failure or ARDS, AKI, and shock (*n* = 54, 20, and 16, respectively, Figure 2). The baseline pre- and perioperative patient characteristics are summarized in Table 1. In brief, patients with MPC had greater body mass index, more hypertension, and worse NYHA cardiac functional classification at baseline. Preoperative blood gas analysis showed no significant difference between the MPC and no-MPC groups, except for a slightly but significantly lower PaO_2_ and PaO_2_/FiO_2_ ratio in patients with MPC (although both were within the normal range in both groups). Patients with MPC had longer surgery duration [310 (270, 405) vs. 290 (242.5, 338.8) min, *p* = 0.006], but there was no difference in the CBP duration and aortic clamping duration. Patients with MPC had higher SOFA scores at ICU admission [12 (11, 13) vs*.* 11 (10, 12.8), *p* = 0.022]. The median (Q_L_, Q_U_) mechanical ventilation duration was 16 (10, 21) hours, with a longer duration in the MPC group [19 (15, 48) h] compared to the no-MPC group [10 (7, 16) h, *p* < 0.001]. The length of ICU stay was 2 (1, 3) days, with a longer stay in the MPC group [3 (2, 4) days] compared to the no-MPC group [1 (1, 2) day, *p* < 0.001]. All deaths occurred in the ICU, and the mortality rate was 4.9% (*n* = 7).

**Table 1:** Preoperative and intraoperative patient characteristics.

	No major postoperative complications (*n* = 65)	With major postoperative complications (*n* = 77)	All (*n* = 142)	*P*-value
Age (year)	57 (49.3, 62)	56 (49.5, 66)	57 (49.8, 65)	0.771
Female (%)	33 (50.8)	38 (49.4)	71 (50)	0.866
Body mass index	22.4 ± 3.1	23.7 ± 3.2	23.1 ± 3.2	0.022
Coexisting conditions				
Hypertension (%)	6 (9.2)	27 (35.1)	33 (23.2)	<0.001
Diabetes (%)	5 (7.7)	9 (11.7)	14 (9.9)	0.426
COPD (%)	3 (4.6)	1 (1.3)	4 (2.8)	0.234
Atrial fibrillation (%)	3 (4.6)	3 (3.9)	6 (4.2)	>0.999
Neurological disorders (%)	3 (4.6)	4 (5.2)	7 (4.9)	>0.999
NYHA grade				0.048
Grade 1	7 (10.8%)	4 (5.2%)	11 (7.7%)	
Grade 2	20 (30.8%)	17 (22.1%)	37 (26.1%)	
Grade 3	35 (53.8%)	49 (63.6%)	84 (59.2%)	
Grade 4	3 (4.6%)	7 (9.1%)	10 (7%)	
Left ventricle ejection fraction (%)	59 (55, 61)	57 (48.5, 60)	58 (52, 60.3)	0.089
Troponin (ng/ml)	0.01 (0.01, 0.07)	0.02 (0.01, 0.03)	0.01 (0.01, 0.02)	0.122
Nt-pro-BNP (pg/ml)	428.1 (116, 1,346.8)	536 (152, 1,794)	491 (123, 1,612)	0.201
Creatinine (*μ*mol/L)	70 (63, 80.8)	71 (60, 88.5)	71 (60.8, 85.3)	0.555
PH	7.46 ± 0.04	7.46 ± 0.04	7.46 ± 0.04	0.335
PaCO_2_ (mmHg)	39 ± 4.5	39.1 ± 4.2	39 ± 4.3	0.783
PaO_2_ (mmHg)	86 (81, 93)	81 (72, 86)	83 (75, 89)	<0.001
PaO_2_/FiO_2_	407.1 (382.1, 441.7)	385.7 (338.1, 409.5)	392.9 (351.1, 419)	<0.001
Hemoglobin (g/dl)	13.4 ± 1.7	13.2 ± 1.9	13.2 ± 1.8	0.642
Hematocrit (%)	41.7 ± 5.2	40.9 ± 6.1	41.3 ± 5.7	0.500
Lactate (mmol/L)	1.2 (0.8, 1.6)	1 (0.8, 1.7)	1.2 (0.8, 1.6)	0.447
Surgery type				0.691
Valve replacement (%)	47 (72.3%)	42 (54.5%)	89 (62.7%)	
CABG (%)	3 (4.6%)	9 (11.7%)	12 (8.5%)	
Major vascular surgery (%)	4 (6.2%)	13 (16.9%)	17 (12%)	
Other (%)^a^	11 (16.9%)	13 (16.9%)	24 (16.9%)	
Surgery duration (min)	290 (242.5, 338.8)	310 (270, 405)	300 (250, 379.3)	0.006
Cardiopulmonary bypass duration (min)	164.5 (118.3, 188.8)	166 (122.5, 227)	166.5 (120.8, 202.8)	0.206
Aortic clamping duration (min)	109 (78.3, 131.3)	103 (74, 142)	108 (76, 134.5)	0.819
Intraoperative fluid balance (ml)	245 (−322.5, 655)	200 (−440, 871)	200 (−400, 757.5)	0.811
APACHE-II score at ICU adminssion	27.2 ± 6.8	28.8 ± 5.9	28.1 ± 5.5	0.077
SOFA score at ICU admission	11 (10, 12.8)	12 (11, 13)	11 (10, 13)	0.022

APACHE-II, acute physiology and chronic health evaluation-II; CABG, coronary artery bypass grafting; COPD, chronic obstructive pulmonary disease; NYHA, New York Heart Association; Nt-pro-BNP, N-terminal pro-B-type natriuretic peptide; SOFA:, sequential organ failure assessment.

^a^ including congenital heart disease surgery, cardiac tumor resection, and combined cardiac surgery.

**Figure 3:**
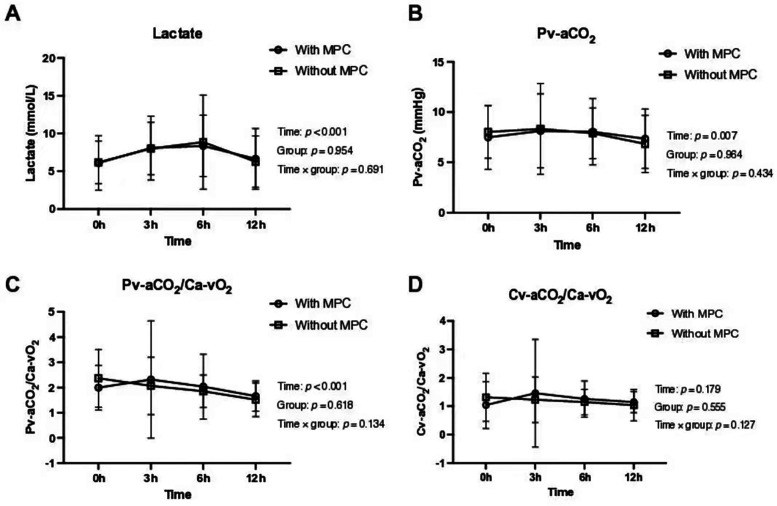
Lactate and CO₂-derived parameters in the first 12 postoperative hours according to major postoperative complications groups. **(A)** Lactate levels over time. **(B)** Venous-arterial difference in CO₂ partial pressure (Pv-aCO₂) over time. **(C)** Venous-arterial difference in CO₂ partial pressure to arterial-venous O₂ content ratio (Pv-aCO₂/Ca-vO₂) over time. **(D)** Venous-arterial difference in CO₂ content to arterial-venous O₂ content ratio (Cv-aCO₂/Ca-vO₂) over time. All parameters were assessed in patients with and without major postoperative complications (MPC).

No statistically significant difference was observed between the MPC and no-MPC groups in terms of lactate and CO_2_-derived parameters. However, a significant difference was observed between the time points, except for Cv-aCO_2_/Ca-vO_2_ (Table 2 and Figure 3). Similar time effects were observed in arterial pH, PaCO_2_, PvCO_2_, arterial and venous blood CO_2_ content, Cv-aCO_2_, and hemoglobin, and no group effect was observed except hemoglobin. On the other hand, O_2_-derived parameters (i.e., PaO_2_, PvO_2_, and PaO_2_/FiO_2_) showed a significant difference between the MPC and no-MPC groups, but there was no intra-group difference in Ca-vO_2_ (Table 2).

**Table 2:** Results of postoperative blood gas analysis.

	0 h		3 h		6 h		12 h		*P*-value 1	*P-*value 2	*P*-value 3
	No MPC (*n* = 65)	With MPC (*n* = 77)	No MPC (*n* = 65)	With MPC (*n* = 77)	No MPC (*n* = 65)	With MPC (*n* = 77)	No MPC (*n* = 65)	With MPC (*n* = 77)			
Arterial pH	7.346 ± 0.067	7.324 ± 0.082	7.323 (7.283, 7.379)	7.323 (7.273, 7.364)	7.342 ± 0.058	7.35 ± 0.065	7.388 ± 0.052	7.402 ± 0.065	<0.001	0.921	0.078
PaCO_2_ (mmHg)	37.5 ± 6.3	41.2 ± 7.9	38.5 ± 5.8	39.4 ± 6.2	37.5 ± 5.6	37.3 ± 5.5	38.7 (35, 41.5)	36.6 (33.4, 39.9)	0.001	0.268	0.001
PaO_2_ (mmHg)	153 (123.5, 192)	105 (87, 145.5)	151 (123.5, 173.5)	107 (89.3, 140.5)	150 (129, 175)	116 (93.4, 132.5)	149 (116.5, 193)	104 (80.6, 119.5)	0.426	<0.001	0.233
PaO_2_/FiO_2_	393 (313, 480)	245 (196, 331)	397 (356, 461)	268 (213, 355)	384 (355, 462)	290 (220, 344)	408 (319, 500)	270 (196, 323)	0.641	<0.001	0.263
PvCO_2_ (mmHg)	45.5 ± 6.6	48.7 ± 7.5	46.8 ± 6.9	47.6 ± 6.9	45.4 ± 6	45.3 ± 6	44.8 ± 6.5	44.1 ± 5.5	<0.001	0.327	0.014
PvO_2_ (mmHg)	46.4 (40.9, 50.4)	44.2 (36.2, 50.3)	44.1 (40.7, 49.8)	43.4 (37.8, 49.7)	43.1 ± 6.1	40.9 ± 7.2	44.1 ± 5.5	37.2 ± 7.2	0.002	0.018	0.551
Arterial blood CO_2_ content (ml/dl)	41.6 (37.7, 44.6)	43.6 (39.7, 47.5)	40.4 ± 5.1	41.8 ± 5.2	40.7 ± 5.8	41.6 ± 5.8	45.3 (42, 49.7)	45.6 (41.1, 50.6)	<0.001	0.059	0.533
Venous blood CO_2_ content (ml/dl)	46 ± 4.4	47.5 ± 5	45.5 (41.1, 48.4)	46.7 (43, 51.1)	45.7 ± 6.1	46.7 ± 6.3	49.9 ± 6.5	50.8 ± 6.5	<0.001	0.08	0.933
Arterial O_2_ content (ml/dl)	15.3 ± 2	15 ± 2.2	16.8 (14.5, 18.2)	15.7 (14.4, 17)	16.6 ± 2.3	15.3 ± 2.3	16.5 ± 2.3	15 ± 2.6	<0.001	0.003	0.003
Venous O_2_ content (ml/dl)	11.6 ± 2.2	10.9 ± 2.6	12.2 (10.5, 14)	11.3 (10, 12.9)	12.2 ± 2.4	10.9 ± 2.7	11 (9.3, 12.7)	10.7 (8.9, 12.1)	<0.001	0.007	0.225
Pv-aCO_2_ (ml)	8 ± 2.6	7.5 ± 3.2	7.8 (6, 10.1)	7.4 (5.7, 10.4)	7.7 (6.7, 9.4)	7.6 (5.7, 9.8)	6.8 ± 2.8	7.4 ± 2.9	0.007	0.964	0.434
Cv-aCO_2_ (ml)	4.4 (3, 5.4)	3.7 (2.1, 5.1)	4.8 (3.2, 6.2)	4.2 (3.3, 6.2)	4.6 (4, 5.7)	4.7 (3.6, 6.2)	4.9 (3.2, 6.1)	4.7 (3.6, 6.3)	0.025	0.926	0.476
Ca-vO_2_ (ml)	3.6 (3, 4.4)	3.6 (2.9, 4.8)	4.4 (3.3, 5)	3.8 (3, 5.3)	4.4 (3.5, 4.9)	4.2 (3.3, 5.2)	4.7 (3.7, 5.7)	4.4 (3.4, 5.5)	<0.001	0.938	0.334
Pv-aCO_2_/Ca-vO_2_ (ml)	2.2 (1.7, 2.7)	1.9 (1.4, 2.5)	1.8 (1.4, 2.3)	1.9 (1.6, 2.4)	1.8 (1.5, 2.2)	1.9 (1.5, 2.2)	1.4 (1, 1.9)	1.6 (1.2, 2)	<0.001	0.618	0.134
Cv-aCO_2_/Ca-vO_2_ (ml)	1.1 (0.9, 1.4)	0.9 (0.6, 1.3)	1.1 (0.7, 1.4)	1.1 (0.9, 1.4)	1.1 (0.9, 1.4)	1.1 (0.9, 1.4)	1 (0.7, 1.3)	1.1 (0.9, 1.3)	0.179	0.555	0.127
Hemoglobin (g/dl)	11.2 ± 1.5	11.2 ± 1.6	12.1 ± 1.7	11.6 ± 2	12.2 ± 1.8	11.4 ± 1.8	12 ± 1.8	11.2 ± 2	<0.001	0.047	0.001
Hematocrit (%)	34 (31.3, 37.6)	34.2 (31.4, 37.5)	37.2 ± 5.3	35.6 ± 6	37.4 ± 5.4	34.9 ± 5.6	36.9 ± 5.3	34.5 ± 6.3	0.790	0.581	0.155
Lactate (mmol/L)	5.5 (4.3, 8)	5.1 (3.5, 8.7)	8 ± 3.5	8.1 ± 4.2	8.5 (6.1, 10)	8.6 (5.6, 11.2)	5.6 (3.8, 8.4)	5.8 (3.7, 8.6)	<0.001	0.954	0.691

*P*-value 1 was the significance of the time effect, *p*-value 2 was the significance of the group effect, and *p*-value 3 was the significance of time×group interaction.

MPC, major postoperative complications.

**Figure 4:**
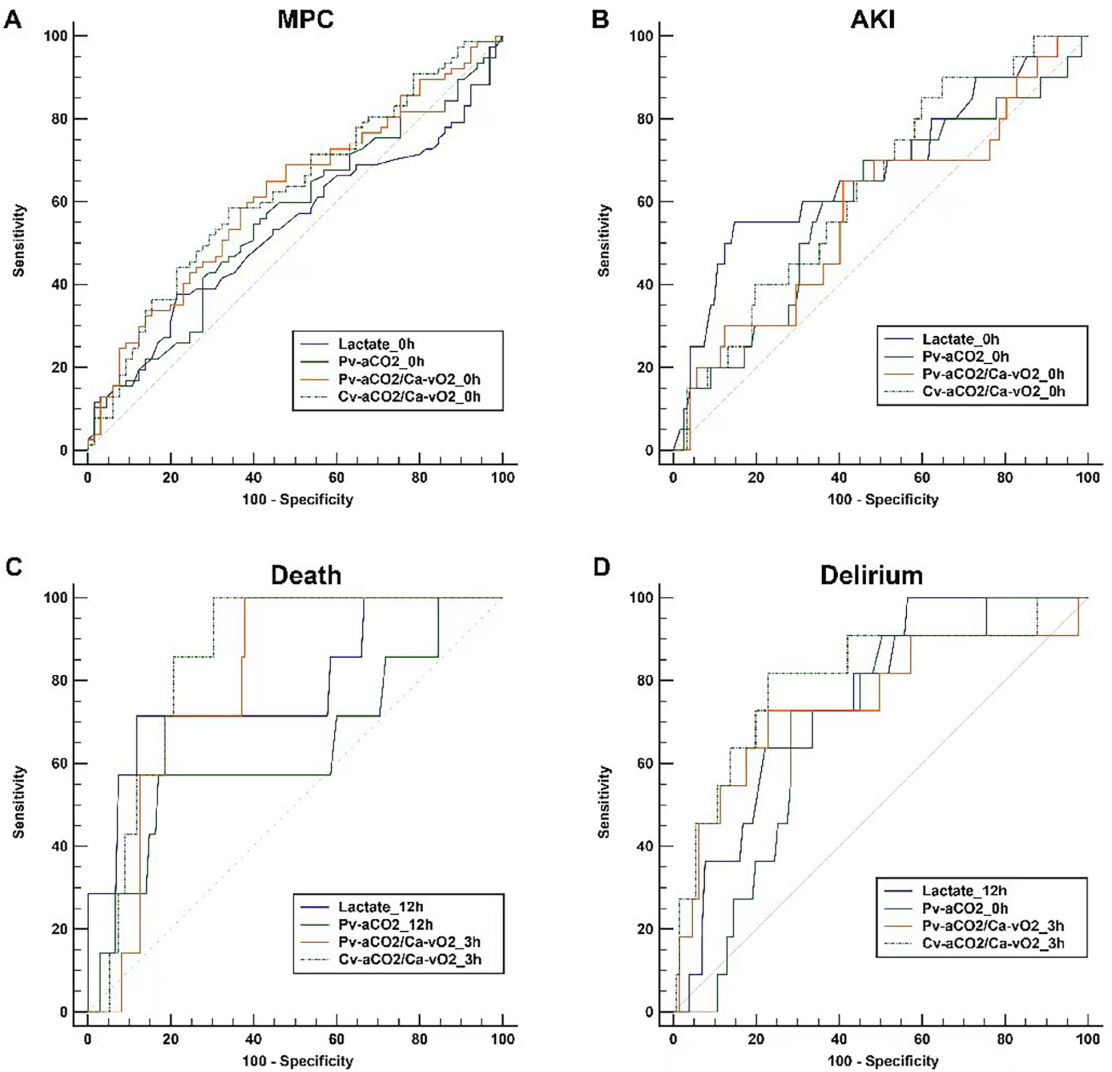
Comparisons of the receiver-operating characteristics curves. For each parameter, only the curve with the highest AUC among different time points is presented. Panel **(A)** Major postoperative complications (MPC) as the outcome; Panel **(B)** Acute renal failure (AKI) as the outcome; Panel **(C)** Death as the outcome; Panel **(D)** Delirium as the outcome.

For the ROC analysis, we only considered the time points with the highest AUCs for comparing different parameters. AUCs of all the time points are presented in the Supplementary File (S1, Table S1). Lactate and CO_2_-derived parameters had the greatest AUCs at 0 h in predicting MPC, and there was no significant difference between these four parameters. Generally, lactate and CO_2_-derived parameters had poor performance in predicting MPC, with AUCs of 0.532 (95% CI: 0.446–0.616) for lactate, 0.559 (0.473–0.642) for Pv-aCO_2_, 0.617 (0.532–0.697) for Pv-aCO_2_/Ca-vO_2_, and 0.625 (0.540–0.705) for Cv-aCO_2_/Ca-vO_2_, respectively (Figure 4A and Table 3).

**Table 3:** Comparisons of receiver-operating characteristics curves of postoperative lactate and CO_2_-derived parameters.

	AUC (95% CI)	Cut-off value	Sensitivity (%, 95% CI)	Specificity (%, 95% CI)	PLR (95% CI)	NLR (95% CI)	PPV (%, 95% CI)	NPV (%, 95% CI)
Major postoperative complications								
Lactate 0 h	0.532 (0.446–0.616)	4.1	37.7 (26.9–49.4)	78.5 (66.5–87.7)	1.75 (1.0–3.0)	0.79 (0.6–1.0)	67.4 (51.5–80.9)	51.5 (41.3–61.7)
Pv-aCO_2_ 0 h	0.559 (0.473–0.642)	7.7	54.6 (42.8–65.9)	60 (47.1–72.0)	1.36 (1.0–2.0)	0.76 (0.6–1.0)	61.8 (49.2–73.3)	52.7 (40.7–64.4)
Pv-aCO_2_/Ca-vO_2_ 0 h	0.617 (0.532–0.697)	2.1	64.9 (53.2–75.5)	56.9 (44.0–69.2)	1.51 (1.1–2.1)	0.62 (0.4–0.9)	64.1 (52.4–74.7)	57.8 (44.8–70.1)
Cv-aCO_2_/Ca-vO_2_ 0 h	0.625 (0.540–0.705)	1	58.4 (46.6–69.6)	66.2 (53.4–77.4)	1.73 (1.2–2.5)	0.63 (0.5–0.9)	67.2 (54.6–78.2)	57.3 (45.4–68.7)
Acute kidney failure								
Lactate 0 h	0.678 (0.595–0.754)	8.6	55 (31.5–76.9)	85.3 (77.7–91.0)	3.73 (2.1–6.7)	0.53 (0.3–0.9)	37.9 (20.7–57.7)	92 (85.4–96.3)
Pv-aCO_2_ 0 h	0.593 (0.507–0.674)	7.7	70 (45.7–88.1)	54.1 (44.8–63.2)	1.53 (1.1–2.2)	0.55 (0.3–1.1)	20 (11.4–31.3)	91.7 (82.7–96.9)
Pv-aCO_2_/Ca-vO_2_ 0 h	0.577 (0.491–0.659)	1.9	65 (40.8–84.6)	59 (49.7–67.8)	1.59 (1.1–2.3)	0.59 (0.3–1.1)	20.6 (11.5–32.7)	91.1 (82.6–96.4)
Cv-aCO_2_/Ca-vO_2_ 0 h	0.635 (0.550–0.714)	1.3	90 (68.3–98.8)	35.3 (26.8–44.4)	1.39 (1.1–1.7)	0.28 (0.07–1.1)	18.6 (11.4–27.7)	95.6 (84.9–99.5)
Death								
Lactate 12 h	0.785 (0.708–0.850)	10.1	71.4 (29.0–96.3)	88.2 (81.5–93.1)	6.03 (3.1–11.6)	0.32 (0.1–1.0)	23.8 (8.2–47.2)	98.3 (94.2–99.8)
Pv-aCO_2_ 12 h	0.635 (0.550–0.714)	9.8	57.1 (18.4–90.1)	83 (75.5–88.9)	3.35 (1.6–7.0)	0.52 (0.2–1.2)	14.8 (4.2–33.7)	97.4 (92.6–99.5)
Pv-aCO_2_/Ca-vO_2_ 3 h	0.801 (0.726–0.863)	2	100 (59.0–100.0)	62.2 (53.5–70.4)	2.65 (2.1–3.3)	0 (0–0)	12.1 (5.0–23.3)	100 (95.7–100.0)
Cv-aCO_2_/Ca-vO_2_ 3 h	0.853 (0.784–0.907)	1.3	100 (59.0–100.0)	69.6 (61.1–77.2)	3.29 (2.6–4.3)	0 (0–0)	14.6 (6.1–27.8)	100 (96.2–100.0)
Delirium								
Lactate 12 h	0.756 (0.677–0.824)	5.2	100 (71.5–100.0)	43.5 (34.9–52.4)	1.77 (1.5–2.1)	0 (0–0)	12.9 (6.6–22.0)	100 (93.7–100.0)
Pv-aCO_2_ 0 h	0.694 (0.611–0.768)	6.1	72.7 (39.0–94.0)	71.8 (63.2–79.3)	2.57 (1.6–4.1)	0.38 (0.1–1.0)	17.8 (8.0–32.1)	96.9 (91.2–99.4)
Pv-aCO_2_/Ca-vO_2_ 3 h	0.749 (0.669–0.818)	2.3	72.7 (39.0–94.0)	77.1 (68.9–84.0)	3.18 (2.0–5.1)	0.35 (0.1–0.9)	21.1 (9.6–37.3)	97.1 (91.8–99.4)
Cv-aCO_2_/Ca-vO_2_ 3 h	0.808 (0.733–0.869)	1.4	81.8 (48.2–97.7)	77.1 (68.9–84.0)	3.57 (2.3–5.4)	0.24 (0.07–0.8)	23.1 (11.1–39.3)	98.1 (93.2–99.8)

AUC, area under the curve; CI, confidence interval; NLR, negative likelihood ratio; NPV, negative predictive value; PLR, positive likelihood ratio; PPV, positive predictive value.

Each parameter was analyzed for its highest AUC across the four time points. In pairwise comparisons, no significant difference was observed in major postoperative complications, acute renal failure, or death. The AUC of Cv-aCO_2_/Ca-vO_2_ was significantly greater than the AUC of Pv-aCO_2_/Ca-vO_2_ (*p* = 0.042) in predicting delirium, while other pairwise comparisons yield no significant difference.

In addition, we conducted a *post-hoc* analysis to examine how well lactate and CO_2_-derived parameters can predict MPC components that are more likely related to tissue hypoxia, including AKI, delirium, and death. Lactate levels in patients with AKI significantly differed over time and between groups. Pv-aCO_2_ levels only had a significant difference over time, while no significant differences were observed in Pv-aCO_2_/Ca-vO_2_ and Cv-aCO_2_/Ca-vO_2_ between groups or over time (Supplementary File S1, Figure S1). In the ROC analysis, all parameters had the greatest AUCs at 0 h in predicting AKI. However, all AUCs were lower than 0.75, and these parameters had no significant difference (Table 3 and Figure 4B).

With death as the grouping factor, lactate significantly changed over time (time effect), while no group effect or time×group interaction was observed. No significant time or group effect was observed in Pv-aCO_2_, Pv-aCO_2_/Ca-vO_2_, or Cv-aCO_2_/Ca-vO_2_ (Supplementary File S1, Figure S2). In the ROC analysis, Pv-aCO_2_/Ca-vO_2_ and Cv-aCO_2_/Ca-vO_2_ at 3 h, and lactate and Pv-aCO_2_ at 12 h had the greatest AUCs in predicting mortality (Figure 4C). Cv-aCO_2_/Ca-vO_2_ yielded the highest AUC of 0.853 (95% CI: 0.784–0.907) among these parameters with a cut-off value of 1.3, the sensitivity was 100% (95% CI: 59.0–100.0%), and the specificity was 69.6% (95% CI: 61.1–77.2%); however, the difference of the AUCs did not reach the significance level (Table 3). With delirium as the grouping factor, a significant time effect was observed in all these four parameters. Meanwhile, a significant group effect and time×group interaction was observed in Cv-aCO_2_/Ca-vO_2_ (Supplementary File S1, Figure S3). In the ROC analysis, Pv-aCO_2_/Ca-vO_2_ and Cv-aCO_2_/Ca-vO_2_ at 3 h, lactate at 12 h, and Pv-aCO_2_ at 0 h had the greatest AUCs in predicting delirium (Figure 4D). Cv-aCO_2_/Ca-vO_2_ yielded the highest AUC of 0.808 (95% CI: 0.733–0.869) among these parameters with a cut-off value of 1.4, the sensitivity was 81.8% (95% CI: 48.2–97.7%), and the specificity was 77.1% (95% CI: 68.9–84.0%); a significant difference was observed between the AUCs of Pv-aCO_2_/Ca-vO_2_ and Cv-aCO_2_/Ca-vO_2_ (*p* = 0.042, Table 3).

Hypertension was significantly associated with the outcome across all models, with hazard ratios (HRs) ranging from 2.03 (95% CI: 1.45–13.89, *p* = 0.001) in Model 1 to 1.40 (95% CI: 1.40–11.21, *p* = 0.01) in Model 3. Surgery Duration also showed a consistent association, with HRs of 1.002 (95% CI: 1.002–1.011, *p* = 0.003) in Model 1 and 1.001 (95% CI: 1.001–1.011, *p* = 0.015) in Model 3. PaO_2_/FiO_2_ was significantly predictive in all models, with HRs of 0.98 (95% CI: 0.98–0.994, *p* = 0.001) in Model 1 and 0.982 (95% CI: 0.982–0.997, *p* = 0.007) in Model 3. Lactate 0 h and CO_2_-derived parameters (e.g., Pv-aCO_2_/Ca-vO_2_ 0 h) did not show significant associations in Models 2 and 3 (*p* > 0.05), except for Pv-aCO_2_/Ca-vO_2_ 0 h in Model 1 (HR: 0.45, 95% CI: 0.45–0.98, *p* = 0.039, Table 4).

**Table 4:** Univariate and multivariate modeling for prediction of major postoperative complications.

Variable	Univariate analysis		Multivariate model 1		Multivariate model 2	
	95% Confidence interval (CI)	*P*-value	95% Confidence interval (CI)	*P*-value	95% Confidence interval (CI)	*P*-value
Hypertension	2.030–13.889	0.001	1.399–11.211	0.010	1.453–13.199	0.009
BMI	1.016–1.270	0.025				
Left ventricle ejection fraction	0.919–1.000	0.051				
NYHA grade	0.938–3.829	0.075				
Surgery Duration	1.002–1.011	0.003	1.001–1.011	0.015	1.002–1.014	0.008
SOFA score at ICU admission	1.043–1.416	0.013				
APACHE-II score at ICU adminssion	0.994–1.126	0.079				
Troponin	0.590–5.776	0.292				
PaO_2_/FiO_2_	0.980–0.994	0.001	0.982–0.997	0.007	0.982–0.998	0.010
Lactate 0 h	0.898–1.100	0.905			0.783–1.045	0.174
Pv-aCO_2_ 0 h	0.838–1.054	0.286			0.842–1.187	0.999
Pv-aCO_2_/Ca-vO_2_ 0 h	0.448–0.980	0.039			0.440–1.536	0.539
Cv-aCO_2_/Ca-vO_2_ 0 h	0.387–1.041	0.072			0.498–1.557	0.661

Univariate analysis: Logistic regression evaluating the independent association of each variable with postoperative complications without adjustment for covariates. Multivariate model 1: Multivariate logistic regression using forward stepwise selection method, with variables entering the model at *p* < 0.05 and being removed at *p* > 0.10. This model identified hypertension, surgery duration, and PaO₂/FiO₂ ratio as significant independent predictors. Multivariate model 2: Multivariate logistic regression with forced entry of CO₂-derived parameters (Pv-aCO₂ 0 h, Pv-aCO₂/Ca-vO₂ 0 h, and Cv-aCO₂/Ca-vO₂ 0 h) and lactate while adjusting for the significant covariates identified in Multivariate model 1 (hypertension,.

surgery duration, and PaO₂/FiO₂ ratio). Values represent odds ratios with 95% confidence intervals. Empty cells indicate variables that did not meet significance criteria for inclusion in the respective multivariate models.

APACHE-II, acute physiology and chronic health evaluation II; BMI, body mass index; Ca-vO₂, arterial-venous oxygen content difference; CI, confidence interval; Cv-aCO₂, venous-arterial carbon dioxide content difference; ICU, intensive care unit; NYHA, New York Heart Association; PaO₂/FiO₂, ratio of arterial oxygen partial pressure to fractional inspired oxygen; Pv-aCO₂, venous-arterial carbon dioxide partial pressure difference; SOFA, sequential organ failure assessment.

## Discussion

The main findings of the present study were: (1) there was no difference in lactate and CO_2_-derived parameters between patients with or without MPC, and these parameters had limited predictive performance; (2) the performance of lactate and CO_2_-derived parameters improved in predicting delirium and death; (3) Cv-aCO_2_/Ca-vO_2_ had the best predictive performance in predicting delirium and mortality.

Complications after cardiac surgery are associated with high morbidity and mortality (26–28). Although multifactorial, one crucial factor of postoperative organ dysfunction is the mismatch of oxygen delivery and consumption (29, 30). The imbalance of oxygen delivery and consumption leads to tissue hypoxia, which eventually leads to organ injury and dysfunction if uncorrected. In this regard, parameters reflecting tissue hypoxia may help manage patients undergoing cardiac surgery, and there is increasing interest in these indicators of hypoxia. The majority of previous studies focused on Pv-aCO_2_/Ca-vO_2_ (31–34). Cv-aCO_2_/Ca-vO_2_ was investigated in one prospective observational study involving adult postoperative cardiac surgery patients. However, the aim was to evaluate the ability of Cv-aCO_2_/Ca-vO_2_ and Pv-aCO_2_/Ca-vO_2_ to predict an increase in oxygen consumption upon fluid challenge rather than organ dysfunctions or clinical outcomes (27). To the best of our knowledge, this study is the first to compare the performance of Cv-aCO_2_/Ca-vO_2_ and Pv-aCO_2_/Ca-vO_2_ in predicting outcomes directly.

We found no difference in lactate and CO_2_-derived parameters between patients with or without MPC, and these parameters had limited performance in predicting MPC. One potential explanation is the complex relationship between MPC and anaerobic metabolism. An impaired function of oxygenation of the lung (for example, ARDS or respiratory failure, which is the component of MPC) or an impaired ability of the heart to deliver blood flow to tissues (e.g., shock, circulatory failure, etc.) is the cause of the imbalance of oxygen delivery and consumption rather than the result.

Our finding is consistent with recently published studies (32, 34). A retrospective study found that Pv-aCO_2_ was associated with postoperative adverse outcomes but showed poor diagnostic performance (34). Zhang and colleagues found that Pv-aCO_2_ and Pv-aCO_2_/Ca-vO_2_ cannot be used as reliable indicators to predict the occurrence of organ dysfunction in a prospective observational study (32). In contrast to these negative findings, Mukai et al. reported that postoperative Pv-aCO_2_ was an independent predictor of major organ morbidity and mortality with an AUC of 0.804 (95% CI: 0.688–0.921) (35). However, it is worth noting that the measurements were obtained right at the end of the surgery in their study, which may be a reason for the inconsistent findings. In addition, selected outcomes vary from one study to the other, which is another factor of the discrepancy.

We conducted a *post-hoc* analysis using mortality, AKI, and delirium as the outcome, respectively. The reason for choosing these outcomes was that they are more likely the consequences than the cause of tissue hypoxia. We hypothesized that biomarkers of tissue hypoxia and anaerobic metabolism could have better performance in predicting the consequences of tissue hypoxia. Biomarkers are considered to have good discriminative performance when the AUC is greater than 0.75 (36). Our data suggest that lactate, Pv-aCO_2_/Ca-vO_2_, and Cv-aCO_2_/Ca-vO_2_ had good performance in discriminating mortality and delirium. However, statistical significance was not observed in pairwise comparisons except for the comparison between Pv-aCO_2_/Ca-vO_2_ and Cv-aCO_2_/Ca-vO_2_ in delirium. This finding could be attributed to the limited statistical power of the test, as the number of positive cases was small. On the other hand, lactate and CO_2_-derived parameters had limited performance in predicting AKI. Post-cardiac-surgery AKI is multifactorial, with the spectrum of insults involving micro-embolism, neurohormonal activation, endogenous and exogenous nephrotoxins, metabolic and hemodynamic factors, inflammation, ischemia-reperfusion injury, and oxidative stress (37). A single biomarker of tissue hypoxia may be insufficient to predict AKI.

One aim of the study was to compare the performance of Pv-aCO_2_/Ca-vO_2_ and Cv-aCO_2_/Ca-vO_2._ Our data demonstrate that although Cv-aCO_2_/Ca-vO_2_ had numerically higher AUCs than Pv-aCO_2_/Ca-vO_2_ in predicting MPC, AKI, and mortality, these differences did not reach statistical significance. The only statistically significant difference was observed in predicting delirium (*p* = 0.042). These findings should be interpreted with caution, particularly for the non-significant differences, which should not be over-interpreted as evidence of superiority. The clinical relevance of these statistical differences also warrants consideration. Even for the statistically significant difference observed in predicting delirium, the actual clinical benefit of using Cv-aCO_2_/Ca-vO_2_ over Pv-aCO_2_/Ca-vO_2_ may be modest. While the calculation of Cv-aCO_2_/Ca-vO_2_ does not require additional blood samples compared to Pv-aCO_2_/Ca-vO_2,_ the minimal improvement in predictive performance needs to be balanced against the added complexity of calculation in routine clinical settings. Further studies with larger sample sizes are needed to confirm whether the observed trends in favor of Cv-aCO_2_/Ca-vO_2_ represent clinically meaningful advantages over Pv-aCO_2_/Ca-vO_2,_ and to establish whether these differences are substantial enough to warrant changes in clinical practice.

We utilized a composite endpoint (MPC) in our study. This approach has both strengths and limitations. First, composite endpoints are well-established in cardiovascular research as they capture the overall burden of morbidity, which is particularly relevant in cardiac surgery populations where patients often experience multiple complications with shared underlying mechanisms of tissue hypoperfusion and oxygen supply-demand imbalance (6). Second, composite endpoints provide greater statistical efficiency when individual complications may be relatively uncommon, enhancing statistical power while maintaining clinical relevance. The composite endpoint enhances statistical efficiency and captures the overall burden of morbidity, which is clinically relevant in the cardiac surgical setting where tissue hypoperfusion may manifest in multiple organ systems. However, the heterogeneity within our composite endpoint should be considered when interpreting our findings. The stronger associations observed for certain complications suggest that lactate and CO_2_-derived parameters may be particularly useful for predicting and potentially preventing specific complications rather than the entire spectrum of postoperative morbidity. Future research may benefit from more targeted approaches focusing on homogeneous pathophysiological pathways or organ-specific complications, potentially enhancing both biological plausibility and clinical application of these parameters.

The multivariate analysis identified three independent predictors of MPC after cardiac surgery: preexisting hypertension, extended surgery duration, and reduced PaO₂/FiO₂ ratios. These associations persisted after rigorous adjustment for confounding variables, suggesting their robust prognostic value. Interestingly, while CO₂-derived parameters—specifically Pv-aCO₂/Ca-vO₂ ratio—demonstrated potential predictive capacity in univariate analysis, they failed to maintain statistical significance in multivariate models. This pattern of attenuated association after covariate adjustment suggests that tissue hypoxia biomarkers may reflect downstream consequences of other pathophysiological processes rather than serving as independent causal factors for MPC.

The loss of statistical significance for CO₂-derived parameters in multivariate models should not entirely negate their clinical relevance. Instead, it suggests that these biomarkers may provide complementary information within specific clinical contexts, particularly when interpreted alongside traditional risk factors. Their limited independent predictive value may reflect the complex, multifactorial nature of postoperative complications, which likely extend beyond tissue hypoxia alone.

There were several limitations of the study. The primary limitation was the sample size issue. While our sample size (*n* = 142) was calculated based on a previously reported major postoperative complication rate of 56.5% and provided adequate statistical power (*β* = 0.1, power 90%) for evaluating the ability of lactate and CO_2_-derived parameters to predict MPC, it was evidently insufficient for rare secondary outcomes such as mortality (*n* = 7). This creates a risk of type II errors and, consequently, results regarding these secondary outcomes should be interpreted with caution and considered primarily hypothesis-generating rather than definitive conclusions. The original sample size calculation was based on an expected area under the ROC curve (AUC) of 0.7, a type I error probability (*α*) of 0.05, a type II error probability (*β*) of 0.1, and an anticipated incidence rate of approximately 56.5% for the primary outcome (18). This calculation was appropriate for analyzing the primary outcome but inadequate for secondary endpoints with lower incidence rates. Future research should consider larger multi-center studies to validate our findings, particularly those related to rare outcomes such as mortality. Second, although one important purpose of the study was to compare the performance of the surrogates of the respiratory quotient, no “gold standard” method for measuring respiratory quotient, such as indirect calorimetry (38), was used. Third, central venous rather than mixed venous blood samples were taken to calculate all the CO2-derived parameters. Fourth, the current study was a single-center study, which limits the external validity of the results.

## Conclusions

Lactate and CO_2_-derived parameters including Pv-aCO_2_, Pv-aCO_2_/Ca-vO_2_, and Cv-aCO_2_/Ca-vO_2_ cannot be used as reliable indicators to predict the occurrence of MPC after CBP. Instead, traditional clinical factors such as hypertension, extended surgical duration, and impaired oxygenation emerged as the most reliable risk indicators. Future research should focus on developing multimodal approaches that combine traditional risk factors with novel biomarkers to better identify patients at risk for specific complications in cardiac surgical populations.

## Data Availability

The raw data supporting the conclusions of this article will be made available by the authors, without undue reservation.
