# Supplementary material for: Lactate and CO2-derived parameters are not predictive factors of major postoperative complications after cardiac surgery with cardiopulmonary bypass: a diagnostic accuracy study
Source: Front Cardiovasc Med. 2025 Apr 11;12:1504431. doi: 10.3389/fcvm.2025.1504431 (PMC12022843; doi:10.3389/fcvm.2025.1504431)
Supplement: Supplementary file 1 [file Datasheet1.pdf]

LACTATE AND CO_2-DERIVED PARAMETERS ARE NOT PREDICTIVE FACTORS OF MAJOR POSTOPERATIVE COMPLICATIONS AFTER CARDIAC SURGERY WITH CARDIOPULMONARY BYPASS: A DIAGNOSTIC ACCURACY STUDY

Xiao-Fen Zhou; Han Chen; Jun Ke; Shi-Rong Lin; Ting-Feng Huang; Bing-Ying Chen; Xin-Da Jiang; Feng Chen

Supplementary File

Supplementary Methods

Formulas and Calculation

According to Fick's equation, we have:

$$VO_2 = CO \times (CaO_2 - CvO_2) - \text{Eq. 1}$$

$$VCO_2 = CO \times (CvCO_2 - CaCO_2) - \text{Eq. 2}$$

Where VO_2 is O_2 consumption, CO is cardiac output, CaO_2 is arterial O_2 content, CvO_2 is venous O_2 content, VCO_2 is CO_2 production, $CaCO_2$ is arterial CO_2 content, $CvCO_2$ is venous CO_2 content.

From Eq. 1 and Eq. 2, we have:

$$\frac{VCO_2}{VO_2} = \frac{(CvCO_2 - CaCO_2)}{(CaO_2 - CvO_2)}$$

VCO_2/VO_2 represents the respiratory quotient, and therefore, the right part of the equation ($Cv\text{-}aCO_2/Ca\text{-}vO_2$) serves as a surrogate of the respiratory quotient.

The following formula can describe the relationship between CO_2 partial pressure and CO_2 content:

$$Pv\text{-}aCO_2 = \kappa \times (CvCO_2 - CaCO_2) - \text{Eq. 3}$$

Combined with Eq. 2, we have:

$$VCO_2 = CO \times (PvCO_2 - PaCO_2)/\kappa$$

Rearrange:

$$(PvCO_2 - PaCO_2) = (VCO_2 \times \kappa)/CO$$

This relationship between CO and PCO_2 gap expresses that, to a given VCO_2 and κ (which will not dramatically change in a short period), the PCO_2 gap inversely correlates with CO.

The O_2 related variables are calculated as follows:

- $CaO_2 = (Hb \times SaO_2 \times 1.34) + (PaO_2 \times 0.0031)$
- $CvO_2 = (Hb \times SvO_2 \times 1.34) + (PvO_2 \times 0.0031)$

CO_2 contents is calculated according to the Douglas formula (1)

• Blood CO_2 content = plasma CO_2 content × (1 – 0.0289 × Hb) ÷ (3.352 – 0.456 × SO_2) × (8.142 – pH)

• Plasma CO_2 content = 2.226 × plasma CO_2 solubility × plasma PCO_2 × $(1 + 10^{pH-pK'})$

• Plasma CO_2 solubility = 0.0307 + 0.00057 × (37 – T) + 0.00002 × $(37 - T)^2$

• pK' = 6.086 + 0.042 × (7.4 – pH) + (38 – T) × (0.00472 + 0.00139 × (7.4 – pH))

1. Douglas AR, Jones NL, Reed JW. Calculation of whole blood CO2 content. *J Appl Physiol (1985)* (1988) 65:473-7.

Supplementary Results

Table S1 AUCs of the parameters at each time point

	AUC	95% CI
**Major postoperative complications**		
Pv-aCO_2_0h	0.559	0.473 to 0.642
Pv-aCO_2_3h	0.513	0.428 to 0.598
Pv-aCO_2_6h	0.503	0.418 to 0.588
Pv-aCO_2_12h	0.545	0.459 to 0.628
Cv-aCO_2/Ca-vO_2_0h	0.625	0.540 to 0.705
Cv-aCO_2/Ca-vO_2_3h	0.53	0.445 to 0.614
Cv-aCO_2/Ca-vO_2_6h	0.55	0.464 to 0.633
Cv-aCO_2/Ca-vO_2_12h	0.583	0.498 to 0.665
Lactate_0h	0.532	0.446 to 0.616
Lactate_3h	0.505	0.420 to 0.590
Lactate_6h	0.504	0.419 to 0.589
Lactate_12h	0.511	0.426 to 0.596
Pv-aCO_2/Ca-vO_2_0h	0.617	0.532 to 0.697
Pv-aCO_2/Ca-vO_2_3h	0.537	0.452 to 0.621
Pv-aCO_2/Ca-vO_2_6h	0.537	0.452 to 0.621
Pv-aCO_2/Ca-vO_2_12h	0.566	0.481 to 0.649
**Acute renal failure**		
Pv-aCO_2_0h	0.593	0.507 to 0.674

Pv-aCO_2_3h	0.539	0.453 to 0.622
Pv-aCO_2_6h	0.537	0.452 to 0.621
Pv-aCO_2_12h	0.513	0.428 to 0.598
Cv-aCO_2/Ca-vO_2_0h	0.635	0.550 to 0.714
Cv-aCO_2/Ca-vO_2_3h	0.617	0.532 to 0.697
Cv-aCO_2/Ca-vO_2_6h	0.513	0.428 to 0.598
Cv-aCO_2/Ca-vO_2_12h	0.589	0.504 to 0.671
Lactate_0h	0.678	0.595 to 0.754
Lactate_3h	0.654	0.569 to 0.731
Lactate_6h	0.607	0.521 to 0.687
Lactate_12h	0.579	0.493 to 0.661
Pv-aCO_2/Ca-vO_2_0h	0.577	0.491 to 0.659
Pv-aCO_2/Ca-vO_2_3h	0.562	0.477 to 0.645
Pv-aCO_2/Ca-vO_2_6h	0.542	0.456 to 0.626
Pv-aCO_2/Ca-vO_2_12h	0.569	0.484 to 0.652
**Death**		
Pv-aCO_2_0h	0.594	0.508 to 0.675
Pv-aCO_2_3h	0.573	0.487 to 0.656
Pv-aCO_2_6h	0.624	0.539 to 0.704
Pv-aCO_2_12h	0.635	0.550 to 0.714
Cv-aCO_2/Ca-vO_2_0h	0.62	0.535 to 0.700
Cv-aCO_2/Ca-vO_2_3h	0.853	0.784 to 0.907

Cv-aCO_2/Ca-vO_2_6h	0.658	0.574 to 0.736
Cv-aCO_2/Ca-vO_2_12h	0.537	0.451 to 0.621
Lactate_0h	0.648	0.564 to 0.726
Lactate_3h	0.541	0.456 to 0.625
Lactate_6h	0.681	0.598 to 0.757
Lactate_12h	0.785	0.708 to 0.850
Pv-aCO_2/Ca-vO_2_0h	0.556	0.470 to 0.639
Pv-aCO_2/Ca-vO_2_3h	0.801	0.726 to 0.863
Pv-aCO_2/Ca-vO_2_6h	0.737	0.656 to 0.807
Pv-aCO_2/Ca-vO_2_12h	0.632	0.547 to 0.711
**Delirium**		
Pv-aCO_2_0h	0.694	0.611 to 0.768
Pv-aCO_2_3h	0.552	0.466 to 0.635
Pv-aCO_2_6h	0.515	0.430 to 0.600
Pv-aCO_2_12h	0.532	0.447 to 0.616
Cv-aCO_2/Ca-vO_2_0h	0.579	0.494 to 0.662
Cv-aCO_2/Ca-vO_2_3h	0.808	0.733 to 0.869
Cv-aCO_2/Ca-vO_2_6h	0.709	0.626 to 0.782
Cv-aCO_2/Ca-vO_2_12h	0.62	0.535 to 0.700
Lactate_0h	0.578	0.493 to 0.661
Lactate_3h	0.52	0.435 to 0.605
Lactate_6h	0.635	0.550 to 0.714

Lactate_12h	0.756	0.677 to 0.824
Pv-aCO_2/Ca-vO_2_0h	0.64	0.555 to 0.719
Pv-aCO_2/Ca-vO_2_3h	0.749	0.669 to 0.818
Pv-aCO_2/Ca-vO_2_6h	0.677	0.594 to 0.753
Pv-aCO_2/Ca-vO_2_12h	0.571	0.485 to 0.654

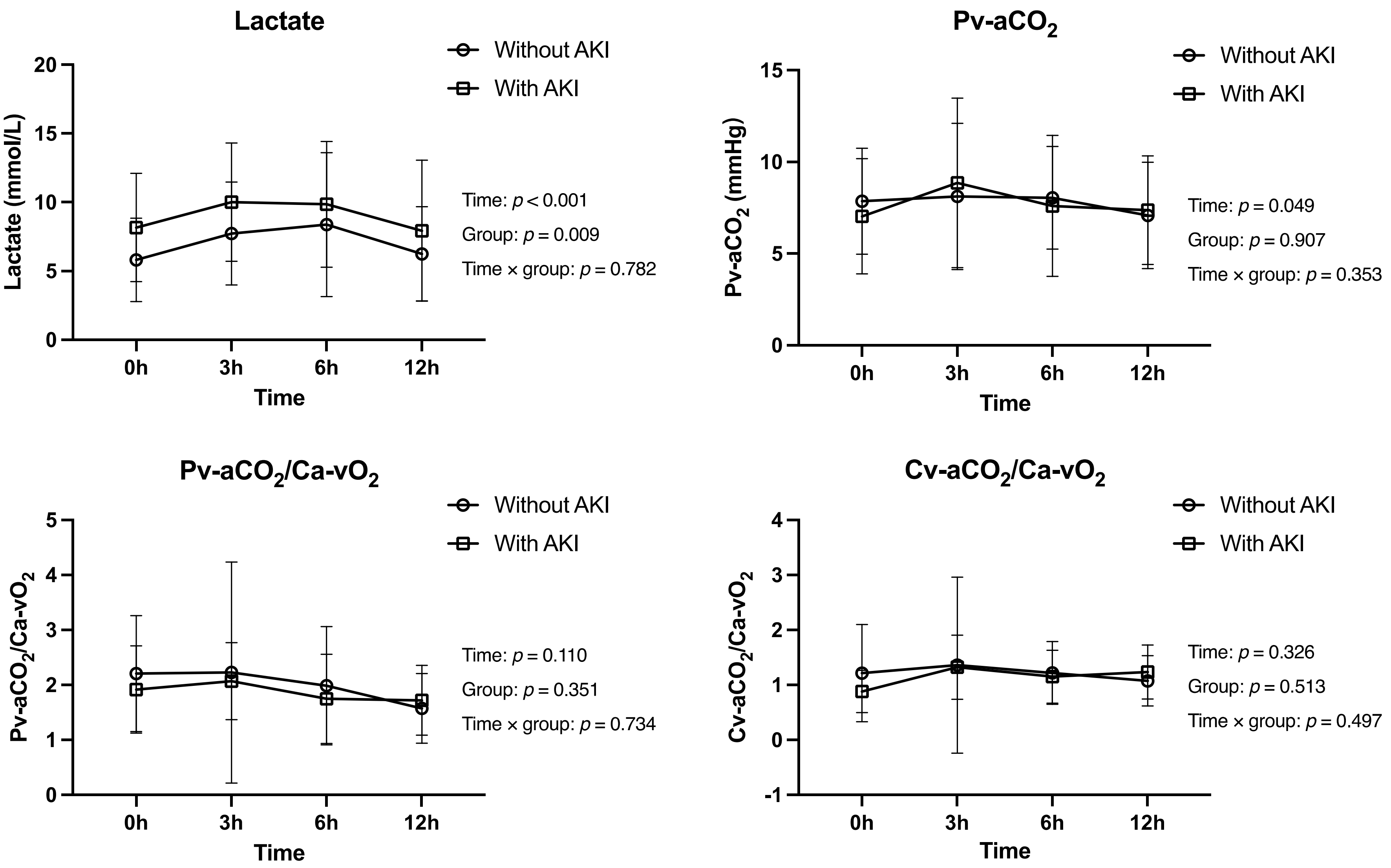

Figure S1 Lactate and CO_2-derived parameters in the first 12 postoperative hours according to acute renal failure

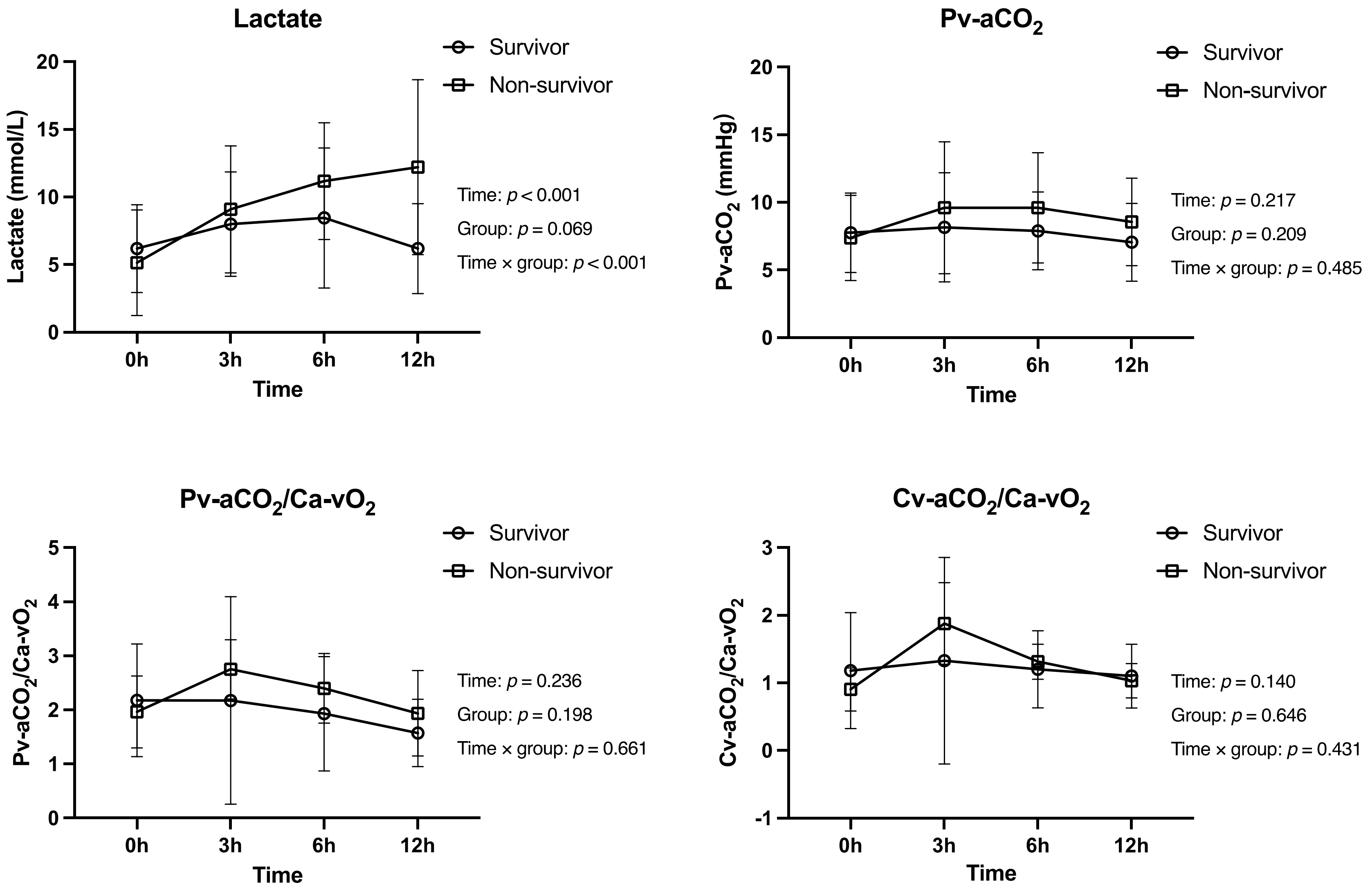

Figure S2 Lactate and CO_2-derived parameters in the first 12 postoperative hours according to mortality.

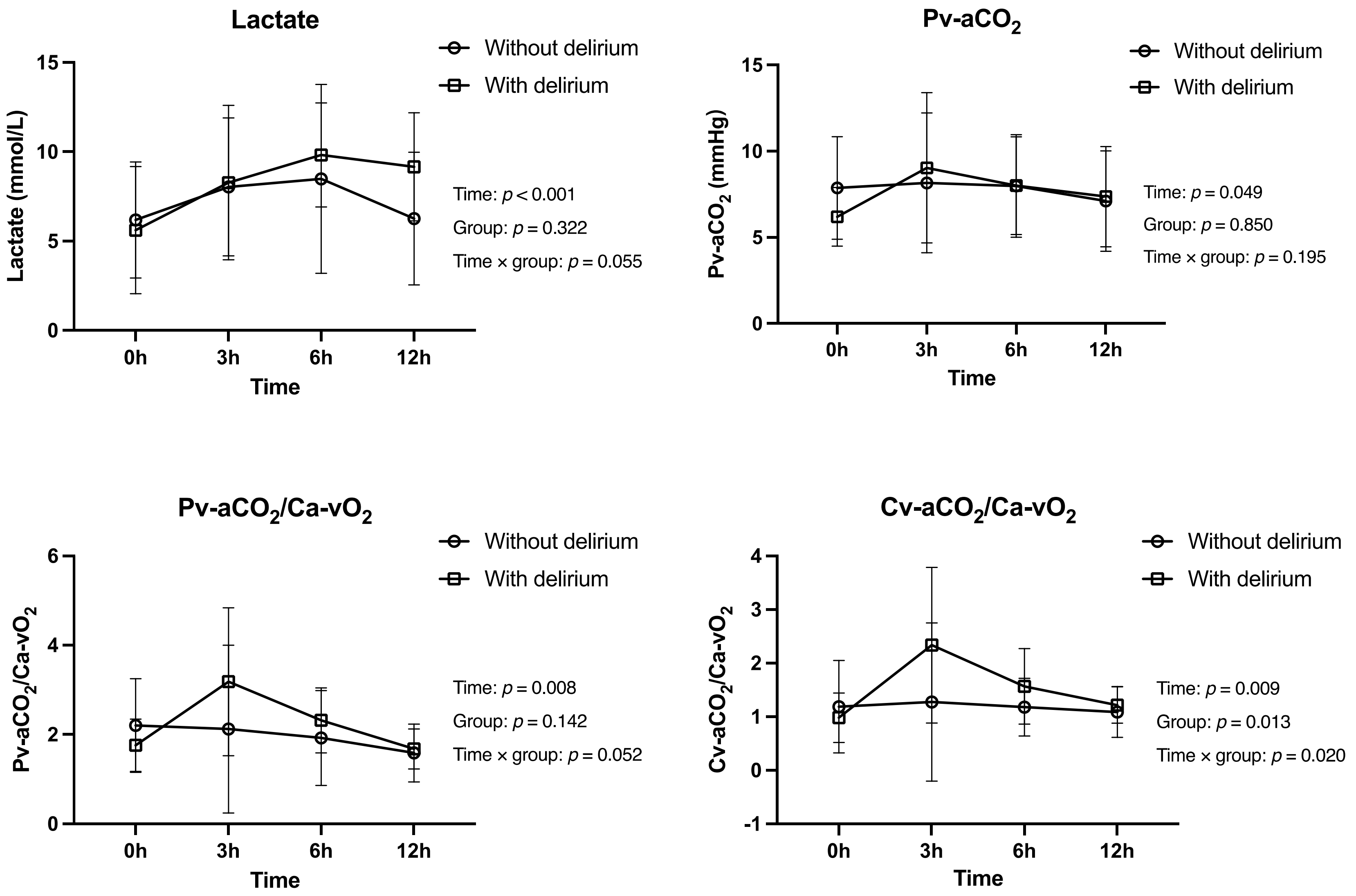

Figure S3 Lactate and CO_2-derived parameters in the first 12 postoperative hours according to delirium.
